# Dysregulation Profile in Preschoolers with Autism Spectrum Disorder: An Italian Multi-Center Perspective

**DOI:** 10.3390/children11121474

**Published:** 2024-11-30

**Authors:** Eugenia Conti, Sara Calderoni, Silvia Guerrera, Andrea Guzzetta, Giuseppina Palermo, Andrea De Giacomo, Raffaella Tancredi, Stefano Vicari, Marco Turi

**Affiliations:** 1 Department of Developmental Neuroscience, IRCCS Fondazione Stella Maris, 56128 Pisa, Italy; eugenia.conti@fsm.unipi.it (E.C.); andrea.guzzetta@fsm.unipi.it (A.G.); raffaella.tancredi@fsm.unipi.it (R.T.); 2 Department of Clinical and Experimental Medicine, University of Pisa, 56126 Pisa, Italy; 3 Child and Adolescent Neuropsychiatry Unit, Bambino Gesù Children’s Hospital, IRCCS, 00165 Rome, Italy; silvia.guerrera@opbg.net (S.G.); stefano.vicari@opbg.net (S.V.); 4 Stella Maris Mediterraneo Foundation, 85032 Chiaromonte, Italy; giusypale6@gmail.com (G.P.); marco.turi@unisalento.it (M.T.); 5 Department of Translational Biomedicine and Neurosciences (DiBraiN), University Hospital, 70124 Bari, Italy; andrea.degiacomo@uniba.it; 6 Life Sciences and Public Health Department, Catholic University, 00153 Rome, Italy; 7 Department of Human and Social Studies, University of Salento, 73100 Lecce, Italy

**Keywords:** Autism Spectrum Disorder, preschoolers, emotional dysregulation, gender, young autistic children

## Abstract

**Background/Objectives**: Autism Spectrum Disorder (ASD) is a lifelong neurodevelopmental condition characterized by social communication impairments and repetitive behaviors. Recent reports show that one in thirty-six 8-year-old children are autistic, signifying a considerable public health concern. According to previous studies, emotional dysregulation (ED) affects 50–60% of individuals with ASD and includes symptoms such as poor emotional control, heightened reactivity, and a low frustration tolerance. The main aims of the current study are to investigate the prevalence of ED among autistic preschoolers (below 6 years of age) and to assess the impacts of gender and intellectual disability on their emotional dysregulation profile. **Methods:** Data have been collected from three children’s research hospitals in Italy (IRCCS Stella Maris Foundation, Stella Maris Mediterraneo Foundation, and IRCCS Bambino Gesù). Parents of 825 autistic pre-schooled children (mean age: 39.68 months, SD: 10.51 months) completed a general questionnaire and the Child Behaviour Checklist (CBCL), a reliable caregiver-reported assessment tool which provides a dysregulation profile. **Results:** A total of 30% of the children displayed a severe-to-moderate ED (emotional dysregulation) profile, with these children exhibiting significantly higher emotional–behavioral problems compared to those without ED. Males with ED exhibited greater emotional reactivity than females with ED. However, no significant relationships were found between ED and age, autism severity (ADOS-2), or intellectual disability. **Conclusions:** The results underline the importance of early, tailored interventions to face emotional challenges in young children with ASD, potentially improving long-term outcomes for this population.

## 1. Introduction

Autism Spectrum Disorder (ASD) is a heterogeneous lifelong condition, characterized by social communication impairment and restricted, repetitive behaviors and interests [1]. A recent analysis published in the CDC’s Morbidity and Mortality Weekly Report reveals that 1 in 36 8-year-old children are considered to have Autism Spectrum Disorder (ASD) [2], thus having a high impact on the public health system.

Alongside the above-mentioned core symptoms, a plethora of associated features are often present in this condition, including emotional dysregulation. Emotional dysregulation (ED) is characterized by poor self-regulation resulting in a low frustration tolerance, irritability, and a high level of emotional reactivity [3,4] and may be detected across diagnostic boundaries. More specifically, poor abstract thinking abilities and impaired psychological mindedness in autistic individuals can lead to poor emotional control, thus having a high impact on their daily life, especially in stressful situations [5].

Recent works report a high prevalence of ED in autistic individuals, varying from 50 to >60%, that impaired emotion regulation is intrinsically linked to Autism Spectrum Disorder, and that altered emotion regulation may underlie or complicate both internalizing and externalizing behavioral disorders [6].

The mutual link between ED and ASD requires research to understand if ED is strictly related to ASD or is related to other comorbidities [7]. Thus, it is important to set up tailored rehabilitative interventions in this heterogeneous clinical population as early as possible [8].

ED has been assessed by previous research through various scales: the Emotion Regulation Questionnaire (ERQ) [9,10,11], the Response to Stress Questionnaire (RSQ) [6,12], the Emotion Regulation Checklist (ERC) [13,14], and the Child Behavior Checklist (CBCL) [15,16]. The latter one, which is a questionnaire compiled by caregivers, has been widely applied, thanks to its availability and feasibility, and includes 113 items grouped into eight syndrome scales. These scores can be grouped into behavioral problem T-scores. The CBCL has been shown to be reliable in clinical and nonclinical populations [17]. A dysregulation profile has also been extrapolated in which high values (above two standard deviations (SDs)) in three syndrome scales (anxiety/depression, attention problems, and aggressive behavior) simultaneously are considered as an indirect measure of emotional dysregulation.

Samson et al. [16] compared fifty-six autistic individuals (47 males and 9 females) and 38 typically developing subjects (26 males and 12 females) between the ages of 6 and 16 years old, reporting greater emotional dysregulation and symptom severity on all scales in the autistic population, specifically in relation to repetitive behaviors. More recently, Joshi and colleagues [5] assessed the prevalence and severity of emotional dysregulation in 123 autistic youth referred for psychiatric comorbidities (with an age range of 5–21 years) compared to children with attention-deficit/hyperactivity disorder (ADHD) (with an age range of 6–18) and controls (with an age range of 6–18). The majority of autistic patients presenting with psychiatric comorbidities had a positive Child Behavior Checklist-ED (CBCL-ED) profile, with scores that were significantly higher than those of the youth with ADHD (82 vs. 53%; *p* < 0.001). Severe emotional dysregulation profiles were significantly greater in the autistic subjects than the ADHD subjects (44 vs. 15%; *p* < 0.001). The ASD youth presenting with a severe emotional dysregulation profile suffered from a greater severity of autism, associated psychopathology, and psychosocial dysfunction. The significant correlation of ED with adaptive functioning alongside global developmental delay and autism symptoms has been recently investigated by Davico et al. [18] in a sample of 100 very young children (74% males with a mean age of 39.4 ± 12.3 months). Though sex differences in emotion regulation competencies have been reported across different phases of development in the general population [19], showing, for example, that females are engaged in more social support-seeking strategies and dysfunctional rumination, whilst males showed more passivity, avoidance, and suppression strategies [20], the authors found no sex differences, probably due to the small sample size investigated. Studies on large sample sizes of children with ASD, e.g., preschoolers, are still lacking.

Wright and colleagues [21] analyzed data from a multicenter longitudinal study on 397 children with ASD aged 2–10 years using the CBCL pre-school and school-age forms and found that anxiety and depression were present in preschoolers, whereas attention problems predominated during late childhood [21].

We present an investigation of the ED distribution, on the basis of the CBCL application, of a wide autistic population of preschoolers (below 6 years of age) referred to three children’s research hospitals in Italy, i.e., IRCCS Stella Maris Foundation (Pisa), Stella Maris Mediterraneo Foundation (Matera), and IRCCS Bambino Gesù Children’s Hospital (Rome), in order to: (i) investigate the prevalence of an emotional dysregulation profile among an autistic sample of preschoolers (below 6 years of age); and (ii) assess the impacts of gender and intellectual disability on the emotional dysregulation profile of this sample.

## 2. Materials and Methods

### 2.1. Participants

The study involved a sample of 825 preschoolers diagnosed with ASD, aged between 18 and 60 months (with a mean age of 39.68 months and SD of 10.51 months), with non-verbal IQ scores ranging from 30 to 137. Of these, 142 were female (17%), with the remainder being male. This subset of participants was drawn from an original cohort of 989 preschool-aged children, including those with and without non-verbal IQ impairments (mean age of 44.0 months, SD of 13.8 months, and age range of 16 to 75 months), who were part of the initial analysis and evaluated in previous studies [22,23]. Participants were recruited between April 2006 and August 2018 from three Italian pediatric care centers: IRCCS Stella Maris Foundation in Pisa, IRCCS Bambino Gesù Children’s Hospital in Rome, and Stella Maris Mediterraneo Foundation in Matera. All children had a primary diagnosis of Autism Spectrum Disorder, with no other known medical or psychiatric comorbidities, except for speech disorders, regulation disorders, and anxiety traits. Diagnoses were established based on DSM-5 criteria [1] or DSM-IV criteria [24] for Autistic Disorder, Asperger’s Disorder, or Pervasive Developmental Disorder—Not Otherwise Specified, due to the retrospective nature of this investigation. A multidisciplinary team, comprising a senior child psychiatrist and an experienced clinical child psychologist, carried out these evaluations. Children with Syndromic Autism, identified causes of ASD, or those who had used psychotropic medication within two months preceding the assessment were excluded. All participants resided in Italy. The study adhered to ethical principles and the Declaration of Helsinki guidelines, with written informed consent obtained from parents or guardians prior to completing the questionnaire.

### 2.2. Measures

#### Autism Diagnostic Observation Scale-G and Autism Diagnostic Observation Scale-2

The Autism Diagnostic Observation Schedule, Second Edition (ADOS-2), is an essential instrument for assessing and diagnosing Autism Spectrum Disorder (ASD). Recognized as the gold standard for autism diagnosis, it employs a structured and standardized method to evaluate social communication, interaction, and play behaviors in individuals suspected of having ASD. The ADOS includes several modules tailored for different age groups and language competencies, facilitating a comprehensive evaluation from toddlers to adults. Each module is crafted to prompt behaviors typical of autism, aiding in accurate diagnosis and intervention planning. In this research, we utilized ADOS-G [25] and ADOS-2 [26]. The ADOS-2 Module of each participant (1 to 4) was chosen according to their age and language development. We converted ADOS-G scores into ADOS-2 scores using the revised algorithm proposed by Gotham et al. [27]. The Calibrated Severity Score (CSS) was computed for each participant based on established algorithms [27,28,29]. Ranging from 1 to 10, the CSS offers an evaluation of ASD symptom severity that is independent of age and language ability, proving to be more suitable than ADOS-2 scores for assessing ASD severity [30].

### 2.3. Cognitive Assessment

To assess the children’s intellectual abilities, several standardized tests were used, tailored to their varying levels of verbal and functional skills. These tests included the Leiter International Performance Scale-Revised (LIPS-R) [31], the Griffiths Mental Development Scales-Extended-Revised (GMDS-ER) [32], and the Italian version of the Wechsler Preschool and Primary Scale of Intelligence (WPPSI) [33]. For tests yielding a mental age (MA), IQ was determined by dividing the mental age by the child’s chronological age (CA) and multiplying the result by 100 (MA/CA × 100). This study specifically concentrated on non-verbal IQ scores, commonly referred to as performance IQ (PIQ).

### 2.4. CBCL 1.5-5

The Italian version of the Child Behavior Checklist for ages 1.5 to 5 years (CBCL 1.5–5) [34] is a widely used and well-established parent reporting tool for evaluating emotional and behavioral problems in young children. This instrument has been validated across diverse cultural settings, proving its reliability and effectiveness in detecting various behavioral concerns, including internalizing and externalizing issues, as well as conditions like ASD [35].

The CBCL 1.5-5 comprises 100 items that parents rate based on their observations of their child’s behavior over the past two months. The responses are categorized into various syndromes and scales, allowing for a comprehensive assessment of a child’s behavioral profile. The CBCL generates scores for seven syndrome scales, three summary scales, and five DSM-oriented scales. This dual structure enhances the tool’s utility in clinical settings, as it aligns with diagnostic criteria while also providing a broader understanding of a child’s behavioral functioning [36]. The Child Behavior Checklist (CBCL) emotional dysregulation profile (CBCL-ED) serves as a crucial tool in identifying emotional and behavioral dysregulation in children and adolescents. This profile is characterized by elevated scores across three specific syndrome scales: anxiety/depression, attention problems, and aggressive behavior. The CBCL-ED is determined by adding the T-scores of the relevant subscales [4]. This cumulative T-score serves as a measure of ED, with higher scores reflecting greater levels of dysregulation. A summed T-score below 180 indicates no ED, scores between 180 and 210 suggest moderate ED, and scores of 210 or higher signify severe ED [37]. The significance of the CBCL-ED lies in its predictive capabilities regarding various psychopathological outcomes, including mood disorders, disruptive behavior disorders, and emotional dysregulation [38,39,40]. Research indicates that children exhibiting high CBCL-ED scores are at an increased risk for developing severe emotional dysregulation, which can manifest in various forms of psychopathology later in life, such as anxiety disorders and bipolar disorder [38,39,41].

### 2.5. Procedure

All participants were clinically diagnosed with ASD and evaluated using the ADOS along with an appropriate psychometric test. Children who could not be assessed with standardized tests due to behavioral issues were excluded. Parents filled out the CBCL at the beginning of the diagnostic process, reflecting their child’s behavior over the past two months or at the present time. For this study, we prioritized the CBCL completed by mothers; if this was not possible, fathers or another close caregiver provided the information. Initially, we analyzed the clinical characteristics of the entire sample. Next, we categorized the whole sample into two groups based on the CBCL-ED score [42]: low emotional dysregulation profile (ED− with a CBCL-ED < 180) and high emotional dysregulation profile (ED+ with CBCL-ED ≥ 180). We then explored differences in gender and intellectual capacity in relation to CBCL-ED distribution between the two groups, comparing their demographics and clinical characteristics.

### 2.6. Data Analysis

All statistical procedures were carried out using IBM SPSS Statistics version 23. We first evaluated the normality of continuous variables by conducting both skewness tests and Kolmogorov–Smirnov tests. For analyzing categorical and continuous independent variables, we used descriptive statistics, chi-square tests, and *t*-tests. To explore potential differences in age, performance IQ (PIQ), CBCL-ED scores, and the various CBCL scales across all groups, we performed independent sample t-tests. A series of two-way between-subject analyses of variance (ANOVAs) were conducted to examine whether group (ED+ or ED−), gender, or intellectual disability (PIQ < 70) had a significant impact on emotional and behavioral problems measured through the CBCL Syndrome scale. Using the statistical software G*Power (Version 3.1.9.6) [43], we assessed the sensitivity of our analyses based on our sample size, a significance level (alpha) of 0.05, and a minimum statistical power of 0.80, following the guidelines proposed by Cohen [44]. This means that our study has an 80% probability of detecting a true effect if it exists and is statistically significant. For the independent t-tests, our calculations indicate that we can reliably detect an effect size of Cohen’s d = 0.21, which represents a small effect. Similarly, for the two-way between-subjects ANOVA conducted in the subgroup analyses, the sensitivity analysis shows that we can detect an effect size of η^2^ = 0.009, which is also considered a small effect. This suggests that our sample size is adequate to identify small but potentially meaningful effects in our data, assuming such effects exist and reach a level of statistical significance. Effect size was calculated based on the statistical method selected for each analysis. For instance, Cohen’s d, eta squared (η^2^), and phi (φ) were used to assess the magnitude of differences between groups or associations between variables, depending on the nature of the analysis.

## 3. Results

As detailed in Table 1, we recruited 825 preschoolers for the study. Using a cut-off score from the CBCL-ED, we divided the sample in two groups: those without an emotional dysregulation profile (ED−) and those with an emotional dysregulation profile (ED+). Following this classification, 69.3% (*n* = 572) of the participants were identified as ED−, while the remaining 30.7% (*n* = 253) were classified as ED+. The average CBCL-ED score across the entire sample was 173.08 (18.32), but it was statistically significantly higher in the ED+ group (M: 194.88, SD: 14.90) compared to the ED− group (M: 163.43, SD: 9.02). Table 1 also indicates that the two groups were comparable in terms of age and PIQ, with no significant differences found (age: *p* = 0.11; PIQ: *p* = 0.21). Additionally, the male-to-female ratio was similar across both groups (*p* = 0.76). Moreover, the analysis of autism symptoms using the ADOS revealed no statistically significant differences between the ED+ and ED− groups in terms of the ADOS total score, social affect (SA), and repetitive restricted behavior (RRB) (ADOS total score: *p* = 0.34; SA: *p* = 0.62; RRB: *p* = 0.28).

Table 2 shows a comparison of CBCL syndrome scale scores between the groups. As anticipated, the ED+ group exhibited consistently higher scores across all CBCL-assessed areas (all *p*-values < 0.0001), reflecting a greater incidence of emotional and behavioral problems linked to this profile.

### 3.1. Sex/Gender Difference

A two-way ANOVA was performed to investigate the effects of sex/gender (S/G) and emotional dysregulation (ED) profile on CBCL Syndrome Scale scores. As showed in Table 3, the analysis found a significant main effect of the ED profile across all CBCL Syndrome Scale scores (all *p* < 0.05), indicating that the children classified as ED+ scored significantly higher on these scales than those classified as ED−.

For S/G, a significant main effect was observed specifically on the Emotionally Reactive scale (F(1,821) = 9.06, *p* = 0.003, η^2^ = 0.01). Males exhibited higher Emotionally Reactive scores (M = 59.85, S.E.M = 0.29) compared to females (M = 57.72, S.E.M = 0.64), regardless of their ED profile. Furthermore, a significant interaction between S/G and group assignment was found for the Emotionally Reactive scale (F(1,821) = 6.48, *p* = 0.01, η^2^ = 0.008) and the Somatic Complaints scale (F(1,821) = 4.12, *p* = 0.04, η^2^ = 0.005). This interaction means that the effect of S/G on these specific scales depends on whether the child is in the ED+ or ED− group. Post hoc tests further revealed that among the ED+ group, the boys had significantly higher scores on the Emotionally Reactive scale compared to girls (males: M = 65.27, SD = 10.02; females: M = 61.36, SD = 9.09; *t*(251) = −2.45, *p* = 0.01, *d* = 0.40). This indicates that the boys with an ED+ profile are particularly prone to emotional reactivity compared to their female counterparts in the same group.

### 3.2. Intellectual Disability

To evaluate the impact of intellectual disability (ID) and emotional dysregulation (ED) profile on CBCL Syndrome Scale scores, a two-way ANOVA was conducted. Within our sample, 33% (*n* = 273) of participants had a PIQ below 70, indicating the presence of an intellectual disability. The distribution of ID was similar across the ED groups, with 33% (*n* = 84) of the ED+ group and 33% (*n* = 189) of the ED− group exhibiting an ID, and no significant difference was observed (χ^2^(1) = 0.002, *p* = 0.96). As shown in Table 4, the analysis revealed a significant main effect of intellectual disability on the Withdrawn scale (F(1,821) = 25.98, *p* < 0.0001, η^2^ = 0.03) and the Total Problems scale (F(1,821) = 10.08, *p* = 0.001, η^2^ = 0.01), indicating that children with an ID scored higher on these scales. However, no significant interaction effects were found between ID and ED profile on any of the CBCL Syndrome Scales (all *p* > 0.05).

## 4. Discussion

Emotional dysregulation is currently of great interest in neuropsychiatry, especially in the autism field in which poor emotional regulation skills have already been detected and potentially impact the functioning of individual preschoolers [45]. ED appears to have significant impacts on global and specific (i.e., social communication) autistic traits, which may lead to important challenges for autistic individuals in psychosocial, behavioral, and cognitive domains [46]. Since ED has been established as a predictor of increased anxiety, suicidality, social isolation, and a reduced overall quality of life in autistic subjects [47], a prompt early identification of this profile is of utmost importance to tailor early interventions and prevent emotional dysregulation at this developmental age. To our knowledge this is the first multi-center study analyzing ED in a huge sample of a well-defined autistic population aged below 6 years. Our work corroborates the feasibility of the CBCL instrument for the investigation of emotional dysregulation characteristics in this young population. According to the previous literature, we divided the available large sample according to the CBCL-ED profile, thus identifying 30.7% (*n* = 253) as being ED+. The low rates of emotional dysregulation (ED) that we found in our work are different from those reported in the literature. Recent studies suggest a high prevalence of ED in autistic populations, ranging from 50% to over 60% [16]. This difference may be due to the instruments and methodologies used to evaluate ED, such as parent reports or self-reports [48], as well as the age range of the participants. However, the current literature on ED in autistic preschool-aged children is still quite limited. In our study, we did not find an association between ED and all areas of autistic symptoms assessed, suggesting that difficulties in emotional regulation are not fully dependent on the core symptoms of autism. This result is consistent with studies in the literature indicating that emotional regulation is not closely related to overall symptom severity [49], although it may be associated with specific ASD symptoms [16]. Specifically, Samson et al. [16] investigated the relationship between parent-reported emotional dysregulation and the core characteristics of autism, finding that only the severity of symptoms in the area of repetitive restricted behaviors was a significant predictor of emotional dysregulation.

We also assessed the psychopathological correlates of the ED profile in the autistic preschoolers using dimensional measures (which evaluate symptoms on a spectrum rather than categorically). The dimensional level of psychopathological dysfunction, as assessed by the Child Behavior Checklist (CBCL), shows that the ED profile influences several syndrome scales. Children in the ED+ group (those with ED) had significantly higher scores compared to the ED− group (those without ED) on the CBCL syndrome scales (“Emotionally Reactive”, “Somatic Complaints”, “Withdrawn”, “Sleep problems”, and “Total Problems”). These findings indicate that children with an ED profile tend to experience more severe emotional and behavioral challenges across multiple areas. Similar results were found by Berkovits et al. [13], who demonstrated that higher levels of emotional regulation competencies were associated with lower scores on the CBCL Total Problems subscale in a group of autistic children, ranging from 4 to 7 years of age, comparable to those evaluated in our study.

In line with this evidence, Favole et al. [50] examined a cohort of 136 children under the age of 6 diagnosed with ASD or other neurodevelopmental disorders (NDD) without ASD. After controlling for factors such as age, intellectual disability (ID)/global developmental delay (GDD), and autism symptom severity, they found a positive correlation between sleep disturbances and ED in both the full sample and within each diagnostic group (ASD and NDD without ASD). Sleep problems were strongly associated with emotional dysregulation in young children with neurodevelopmental disorders, whether or not they had ASD, both cross-sectionally and prospectively. Furthermore, emotional dysregulation was linked to behavioral difficulties, including sleep issues and internalizing problems. These findings suggest that emotional dysregulation may contribute to the early behavioral challenges observed in young children with ASD.

In the present study, the two groups (ED+ vs. ED−) did not differ neither in gender balance, nor in autism severity, letting us keep these two factors aside in the interpretation of the results. The female rate reflected the general epidemiology of the four-to-one male to female distribution of autism, keeping in mind that females are usually underdiagnosed compared to males, due to the mild phenotype, psychiatric comorbidities, and their ability to camouflage, even at comparable levels of autistic symptomatology [51,52,53]. However, our sample is composed of females who received an ASD diagnosis in preschool, and it is possible that the early recognition of these ASD girls is due at least partly to the severity of their clinical presentation. In this framework, a recent systematic review concluded that preschool-age females and males are more similar than different in terms of autistic signs [54]. Indeed, the current investigation also extends the similarities between autistic males and females in the early years of life to ED symptoms. According to this view, the literature suggests that females frequently need additional behavioral problems to be diagnosed as autistic (for a recent systematic review, see [55]). For instance, ASD girls with behavioral difficulties (e.g., hyperactivity) obtain an ASD diagnosis more easily, while boys do not need additional problems to receive an ASD diagnosis [56]. In a similar vein, a large study on 722 participants detected that psychiatrically hospitalized males and females with ASD (age range: 4–20 years) differed, with more severe emotional dysregulation (i.e., higher levels of reactivity and dysphoria) in ASD females than in ASD males [57]. Focusing on preschool-aged children, a clinical and brain imaging study identified that sex/gender differences in psychopathology are already present in toddlers with ASD (age range: 2–4 years), with females presenting a higher level of emotional behavioral problems associated with a larger right amygdala, suggesting a specific brain–behavior correlation [58]. In the general population, females seem to be more competent in activating self-regulatory strategies when experiencing sadness or anxiety [19]. Furthermore, many studies in the literature describing gender differences in terms of phenotype expression and brain correlates in autistic females are available [59,60]. Autistic females are more able than males to inhibit behavioral responses in emotional contexts, related, at a neural level, to the speed of the automatic perceptual processing of facial cues and, in males, to the extent of their active attention allocated to the stimuli [61]. On the other hand, a very recent work from McDonald and colleagues [62] that analyzed the impact of emotional dysregulation in autistic subjects in depth in comparison with neurotypical subjects and other clinical conditions, through a meta-analytic approach, points out a further consideration on the influence of gender in this field. Overall, the authors confirm a moderate magnitude of difference in ED severity between autistic and comparison groups overall, especially vs. neurotypical subjects, though significant heterogeneity was reported across the study samples, warranting moderator analyses. The findings from the moderator analyses suggest a trend toward a greater magnitude of effect size differences, when compared to neurotypical samples with a higher portion of females, which is in opposition with the existing literature (i.e., samples with a higher composition of female participants may experience more severe ED). The authors interestingly theorize that females must often require more autistic traits to receive a formal diagnosis; therefore, it is possible that the female participants included in the final set of studies demonstrated not only more autistic traits but also greater ED, resulting in this surprising finding regarding sex differences in ED.

Investigating the impact of intellectual disability (ID) on the CBCL Syndrome Scale, a main effect of ID was found for the subscales of “Withdrawn” and “Total Problems”. Withdrawal symptoms can be exacerbated in children with intellectual disabilities and Autism Spectrum Disorder. For the effect on the “Total Problems” subscale, sleep disturbances could have a role, too.

In the above-mentioned meta-analysis from McDonald RG and colleagues [62], the authors state that while great significance emerges in the comparison of ED in autistic subjects vs. typical subjects, the effect is small when compared with other clinical conditions, suggesting that severity of ED may be more similar between other clinical conditions and autistic groups. We can then speculate that the co-occurrence of the two conditions could exacerbate clinical impacts on children.

For instance, Saez-Suanes et al. [63] demonstrated that individuals with both ASD and intellectual disability (ID) frequently exhibit considerable depressive symptoms, though the relationship is intricate. Their research indicated that individuals with mild ID often struggle with emotional regulation, resulting in depressive symptoms.

Given the absence of interaction between ID and ED profiles, we suggest that these factors are not closely associated. This finding aligns with the results of Berkovits and colleagues [13], who found that, unlike social and behavioral functioning, emotion regulation in autistic children was not connected to cognitive or language abilities. Notably, 88% of the children in their study had typical IQ scores, suggesting that cognitive ability alone does not protect against challenges in emotional regulation. In typically developing children, certain aspects of self-regulation, particularly those related to attention and executive functioning, are linked to IQ [64]. However, emotion regulation appears to be independent of cognitive abilities, as studies have shown no significant correlations between emotion regulation and IQ, even in typically developing populations (e.g., [65]).

Overall, our data underline the importance of assessing ED characteristics early in autistic populations, considering that together with global developmental delay, ED contributes to impairment in adaptive functioning in childhood [18], in order to set up interventions for the promotion of emotional regulation abilities in this vulnerable population group as early as possible.

Emotional regulation, which develops in the early years of life, is influenced by both intrinsic factors (such as an individual’s ability to regulate their internal emotional responses) and extrinsic factors (such as caregiver interventions). Given this, assessing emotional regulation outcomes in parent-mediated interventions for young children with autism is crucial for customizing early interventions [66]. In early childhood, emotional dysregulation is often understood in terms of irritability (as noted in review [67]) and can manifest in various forms, including externalizing or challenging behaviors. According to Hendrix et al. [66], increasing evidence supports the effectiveness of parent-mediated interventions—in which therapists coach parents to implement coping strategies for their children—in reducing challenging behaviors and enhancing social communication in young children with autism. These interventions may also lead to improved family dynamics, including better parent–child interactions and greater caregiver empowerment. In this context, a longitudinal study involving 186 children with ASD (aged 5 to 12 years) found that individual and family factors (such as a more effective parent–child relationship, improved maternal mental health, and better coping strategies in couples) have a positive influence on the trajectory of emotional dysregulation [68].

## 5. Conclusions

To our knowledge, this is the first multi-center study with a huge sample size to assess emotional dysregulation in the young autistic population. All patients have undergone a multidisciplinary assessment through expert professionals to assess the early diagnosis of autism and related cognitive profiles in the population. The absence of a standardized clinical ED assessment protocol reduces the possibility to interpret results. Future studies including the clinical detection of emotional markers will be useful to enlarge our comprehension of emotional dysregulation phenomena in this delicate young population [49].

However, the CBCL is a widely recognized tool and is easy deliver to parents, giving reliable outputs. A lot of the literature is centered on CBCL assessments, due to its feasibility, thus encouraging its use.

Even though the reliability of parent-reported data has been questioned, due to possible parental bias in interpreting the questions and reliably reporting the characteristics of a certain behavior [69], several studies supported that primary caregivers are usually reliable informants of their child’s early development and everyday functioning [70,71,72] and could also provide important information about behaviors that may not emerge during a circumscribed clinical evaluation [73]. Another limitation of the current study is the lack of adaptive functioning data for all patients, which restricts our ability to conduct meaningful correlation analyses regarding adaptive skills. Additionally, data on parental stress would be valuable. Future longitudinal studies should investigate how both child and family factors influence dysregulation trajectories and examine whether early interventions targeting emotion regulation (ER) lead to changes in core ASD symptoms and related challenges. The ultimate goal is to design tailored interventions.

## Figures and Tables

**Table 1:** Demographic and clinical characteristics in the total sample (*n* = 825) and in each ED group.

	Whole Sample (*n* = 825)	ED− (*n* = 572)	ED+ (*n* = 253)	*t*-Test or *X^2^*	*p*	Effect Size
**Age (months)**						
M (SD)	39.68 (10.51)	39.29 (10.52)	40.55 (10.45)	*t*_(824)_ = −1.59	0.11	*-*
Range	18–60	18–60	19–60			
**Gender (F:M)**	142 (17%):	97 (17%):	45 (18%):	*X*_(1)_ = 0.085	0.76	-
	683 (83%)	475 (83%)	208 (82%)			
**Performance IQ**						
M (SD)	78.54 (22.61)	79.20 (22.50)	77.05 (22.84)	*t*_(824)_ = 1.25	0.21	*-*
Range	17–137	17–137	25–138			
**CSS-Social Affect ADOS**						
M (SD)	6.17 (1.96)	6.15 (1.95)	6.22 (2.02)	*t*_(824)_ = −0.49	0.62	*-*
Range	0–10	1–10	0–10			
**CSS-RRB ADOS**						
M (SD)	7.03 (2.08)	6.98 (2.07)	7.15 (2.10)	*t*_(824)_ = −1.07	0.28	*-*
Range	0–10	1–10	0–10			
**CSS-Total Score ADOS**						
M (SD)	6.32 (1.97)	6.28 (1.95)	6.42 (2.01)	*t*_(824)_ = −0.94	0.34	*-*
Range	0–10	10–1	0–10			
**CBCL-ED**						
M (SD)	173.08 (18.32)	163.43 (9.02)	194.88 (14.90)	*t*_(824)_ = −37.32	<0.0001	*d* = 2.81
Range	133–266	133–179	180–266			

Note. Significant comparisons are highlighted in bold (*p* < 0.05).

**Table 2:** Differences in CBCL syndrome scales between ED+ and ED− groups.

	ED− (*n* = 572)	ED+ (*n* = 253)	*t*-Test	*p*-Value	Effect Size
**Emotional Reactive**					
M (SD)	54.37 (5.57)	64.58 (9.80)	*t*(823) = −18.92	*p* < 0.0001	*d* = 1.42
**Somatic Complaints**					
M (SD)	54.56 (6.12)	60.85 (9.54)	*t*(823) = −11.37	*p* < 0.0001	*d* = 0.85
**Withdrawn**					
M (SD)	66.68 (9.60)	76.52 (9.57)	*t*(823) = −13.57	*p* < 0.0001	*d* = 1.02
**Sleep Problems**					
M (SD)	53.98 (5.76)	60.35 (10.08)	*t*(823) = −11.45	*p* < 0.0001	*d* = 0.86
**Total Problems**					
M (SD)	53.43 (7.87)	69.27 (7.75)	*t*(823) = −26.74	*p* < 0.0001	*d* = 2.02

Note. Comparisons that reached statistical significance are shown in bold (*p* < 0.05).

**Table 3:** Main and interactive effects of ED status, gender, CBCL Syndrome Scale scores.

		Effect of ED Status			Effect of S/G			Effect of Interaction		
	df	F	*p*	η^2^	F	*p*	η^2^	F	*p*	η^2^
**Emotional Reactive**	(1,821)	164.97	**<0.001**	0.16	9.06	**0.003**	**0.01**	6.48	**0.01**	**0.008**
**Somatic Complaints**	(1,821)	53.53	**<0.001**	0.06	1.44	0.23	0.002	4.12	**0.04**	**0.005**
**Withdrawn**	(1,821)	94.31	**<0.001**	0.10	1.89	0.17	0.002	0.91	0.33	0.001
**Sleep Problems**	(1,821)	97.33	**<0.001**	0.10	1.00	0.31	0.001	3.15	0.07	0.004
**Total Problems**	(1,821)	402.41	**<0.001**	0.32	2.75	0.09	0.003	0.21	0.64	0.001

Note. Comparisons that reached statistical significance are shown in bold (*p* < 0.05).

**Table 4:** Main and interactive effects of ED status, ID status, CBCL Syndrome Scale scores.

		Effect of ED Status			Effect of ID Status			Effect of Interaction		
	df	F	*p*	η^2^	F	*p*	Effect Size	F	*p*	η^2^
**Emotional Reactive**	(1,821)	164.97	**<0.001**	0.31	1.13	0.28	0.001	0.01	0.98	0.001
**Somatic Complaints**	(1,821)	53.53	**<0.001**	0.06	1.24	0.26	0.002	1.47	0.22	0.002
**Withdrawn**	(1,821)	94.31	**<0.001**	0.10	**25.98**	**<0.0001**	**0.031**	0.97	0.32	0.001
**Sleep Problems**	(1,821)	97.33	**<0.001**	0.10	0.23	0.62	0.001	1.21	0.27	0.001
**Total Problems**	(1,821)	402.41	**<0.001**	0.32	**10.80**	**0.001**	**0.013**	0.28	0.59	0.001

Note. Comparisons that reached statistical significance are shown in bold (*p* < 0.05).

## Data Availability

Data are available upon request to the authors. The data are not publicly available due to ethical restrictions.
